# Diversity of *Moesziomyces* (Ustilaginales, Ustilaginomycotina) on *Echinochloa* and *Leersia* (Poaceae)

**DOI:** 10.3897/mycokeys.52.30461

**Published:** 2019-05-09

**Authors:** Ying-Ming Li, Roger G. Shivas, Bao-Ju Li, Lei Cai

**Affiliations:** 1 Institute of Vegetables and Flowers, Chinese Academy of Agricultural Sciences, Beijing, 100081, China; 2 Centre for Crop Health, University of Southern Queensland, Toowoomba 4350, Australia; 3 State Key Laboratory of Mycology,; 4 Institute of Microbiology, Chinese Academy of Sciences, Beijing 100101, China

**Keywords:** Ecology, plant pathogens, phylogeny, Ustilaginaceae, Ustilaginomycotina

## Abstract

A combined ecological, morphological, and molecular approach was used to examine 26 herbarium specimens and eight strains of *Moesziomyces*. The phylogenetic analysis resolved eight well-supported clades, of which three contained type specimens of known species of *Moesziomyces*. One clade contained two specimens that produced a teleomorph in the flowers of *Echinochloakimberleyensis* in Australia. The name *Moesziomyceskimberleyensis* is proposed for this smut fungus. Another clade contained specimens that produced sori in the flowers of *Leersiahexandra*. The name *Thecaphoraglobuligera* (now *Moesziomycesglobuligerus*) is available for this species, which is lectotypified. The teleomorph of *Moesziomycesantarcticus*, previously known only from Japan, is found for the first time in China, on *Echinochloacrus-galli*.

## Introduction

The genus *Moesziomyces* (Ustilaginales, Ustilaginaceae) was established by Vánky (1977) for smut fungi that produce sori in the ovaries of grasses, lack a columella, and have spores with irregular meshes and wings on the surface, bound in firmly agglutinated spore balls. Vánky (1977) recognized four species, *M.bullatus*, *M.evernius*, *M.globuligerus*, and *M.penicillariae*. Vánky (1986, 2012, 2013) later synonymised these names with the oldest available name, *M.bullatus*, and considered *Moesziomyces* as monotypic. Species of *Moesziomyces* are known to produce both free-living saprobic anamorphs (yeast-like) and plant pathogenic teleomorphs (smuts) (Wang et al. 2015; Kruse et al. 2017). The anamorphs of *Moesziomyces* are readily culturable on artificial media and have been isolated from a range of substrates, while the teleomorphs are formed in ovaries of seven genera of grasses (Poaceae). Wang et al. (2015) recombined four species known only by their anamorphs (*Pseudozymaantarctica*, *P.aphidis*, *P.parantarctica*, and *P.rugulosa*) into *Moesziomyces*, based on a molecular phylogenetic analysis. Subsequently, Tanaka et al. (2019) showed that one of these species, *M.antarcticus*, produced a teleomorph on *Echinochloacrus-galli* in Japan. A further five species, *M.bullatus*, *M.eriocauli*, *M.evernius*, *M.penicillariae*, and *M.verrucosus*, have been characterized from teleomorphs (Vánky 2012; Wang et al. 2015; Kruse et al. 2017). Kruse et al. (2017) recognized six species of *Moesziomyces* based on phylogenetic analysis, and treated *M.aphidis* and *M.rugulosus* as synonyms of *M.bullatus*.

The teleomorphs of Ustilaginaceae are mostly host specific (Stoll et al. 2003, 2005; Skibbe et al. 2010; McTaggart et al. 2012; Li et al. 2017a, 2017b). Given that species of *Moesziomyces* have been reported from seven different genera of grasses (*Echinochloa*, *Leersia*, *Panicum*, *Paspalum*, *Pennisetum*, *Polytrias*, and *Uranthoecium*), it is likely that additional species remain to be discovered. The aim of this study was to build on the work of Kruse et al. (2017) by examining specimens of *Moesziomyces* held in herbaria BRIP (Queensland Plant Pathology Herbarium), HMAS (Herbarium Mycologicum Academiae Sinicae), and HUV (Herbarium Ustilaginales Vánky, now deposited in BRIP), as well as eight yeast strains deposited in LC Culture Collection (personal culture collection held in the laboratory of Dr Lei Cai).

## Materials and methods

### Specimen examination

Specimens borrowed from several herbaria were examined by light microscopy (Table 1) by mounting the spores in lactic acid (100% v/v). Teliospore measurements were expressed as ranges (min–) mean-standard deviation-mean + standard deviation (–max) (*n* = 50). Images were captured by using a Nikon DS-Fi1 camera attached to a Nikon Eclipse 80i microscope with Nomarski differential interference contrast. Helicon Focus ver. 4.46.1 (Helicon Soft Ltd) was used to combine images to increase depth of field. Nomenclatural novelties and descriptions were registered in MycoBank (http://www.MycoBank.org).

**Table 1.** Collection details for *Moesziomyces* specimens newly sequenced in this study.

Species	Specimen/strain no.^1^	Host	Source	Location	Year of collection	ITS GenBank accession number^2^
* Moesziomyces antarcticus *	HMAS 248025	* Echinochloa crus-galli *	Sorus	China	2017	**MK027038**
* M. antarcticus *	HMAS 248026	* E. crus-galli *	Sorus	China	2017	**MK027039**
* M. antarcticus *	HMAS 60130	* E. crus-galli *	Sorus	China	1989	**MK027043**
* M. bullatus *	HMAS 146471	* E. crus-galli *	Sorus	China	2003	**MK027040**
* M. bullatus *	HMAS 50052	* E. crus-galli *	Sorus	China	1985	**MK027041**
* M. bullatus *	LC-CLS58-3-2	* Setaria faberii *	Leaf surface	China	2017	**MK024201**
* M. bullatus *	LC-CLS58-3-21	* S. faberii *	Leaf surface	China	2017	**MK024202**
* M. bullatus *	LC-CLS58-3-22	* S. faberii *	Leaf surface	China	2017	**MK024203**
* M. bullatus *	LC-CLS60-2-22	*Pennisetum* sp.	Leaf surface	China	2017	**MK024204**
* M. bullatus *	LC-CLS60-2-4	*Pennisetum* sp.	Leaf surface	China	2017	**MK024205**
* M. bullatus *	LC-SY1-2-11	*Digitaria* sp.	Leaf surface	China	2017	**MK024206**
* M. bullatus *	LC-SY1-2-21	*Digitaria* sp.	Leaf surface	China	2017	**MK024207**
* M. bullatus *	LC-SY1-2-22	*Digitaria* sp.	Leaf surface	China	2017	**MK024208**
* M. bullatus *	HMAS 50454	* E. crus-galli *	Sorus	Japan	1985	**MK027042**
* M. bullatus *	HMAS 70876	* E. crus-galli *	Sorus	China	1991	**MK027045**
* M. bullatus *	HMAS 73871	* E. crus-galli *	Sorus	China	1996	**MK027046**
* M. bullatus *	HUV 2442*	* E. crus-galli *	Sorus	Poland	1869	**MK027047**
* M. bullatus *	HUV 305	* E. crus-galli *	Sorus	Germany	1905	**MK027050**
* M. globuligerus *	BRIP 27384	* Leersia hexandra *	Sorus	Australia	1998	**MK027025**
* M. globuligerus *	BRIP 44301	* L. hexandra *	Sorus	Australia	2004	**MK027029**
* M. globuligerus *	BRIP 44569	* L. hexandra *	Sorus	Australia	2004	**MK027030**
* M. globuligerus *	BRIP 47767	* L. hexandra *	Sorus	Thailand	2005	**MK027031**
* M. globuligerus *	BRIP 47768	* L. hexandra *	Sorus	Thailand	2005	**MK027032**
* M. globuligerus *	BRIP 51872	* L. hexandra *	Sorus	Australia	2008	**MK027035**
* M. globuligerus *	HMAS 248027	* L. hexandra *	Sorus	China	2017	**MK027037**
* M. kimberleyensis *	BRIP 51843*	* E. kimberleyensis *	Sorus	Australia	2008	**MK027034**
* M. kimberleyensis *	BRIP 52498	* E. kimberleyensis *	Sorus	Australia	2009	**MK027036**
* M. penicillariae *	HUV 2487	* Pe. glaucum *	Sorus	Gambia	1973	**MK027048**
* M. penicillariae *	HUV 2488	* Pe. glaucum *	Sorus	India	1912	**MK027049**
* M. verrucosus *	BRIP 39886	* Paspalum distichum *	Sorus	Australia	2003	**MK027026**
* M. verrucosus *	BRIP 43727	* Pa. distichum *	Sorus	Australia	2004	**MK027027**
* M. verrucosus *	BRIP 43735	* Pa. distichum *	Sorus	Australia	2004	**MK027028**
* M. verrucosus *	BRIP 51772	* Pa. distichum *	Sorus	India	1992	**MK027033**
* M. verrucosus *	HMAS 66437	* Pa. distichum *	Sorus	India	1992	**MK027044**

^1^BRIP: Queensland Plant Pathology Herbarium, Brisbane, Australia; HMAS: Herbarium Mycologicum Academiae Sinicae; HUV: Herbarium Ustilaginales Vánky (located at BRIP). ^2^GenBank accessions derived from this study are shown in bold. * Type specimens.

### DNA extraction, PCR amplification and sequencing

Sori were carefully removed from herbarium specimens, up to 149 years old, with a fine needle, sterilized by dipping in 75% ethanol for 30 s, air-dried on sterilized filter paper, and deposited in cell lysis solution (CTAB). Pure yeast colonies grown on yeast extract peptone dextrose (YPD) plates were transferred to cell lysis solution directly. Genomic DNA was extracted following the protocol of Cubero et al. (1999). Fragments of internal transcribed spacer rDNA were amplified by PCR with primers M-ITS1/ITS4 (White et al. 1990; Stoll et al. 2003).

PCR amplifications were carried out in 25 μl reactions containing 1 μl of genomic DNA template, 9.5 μl distilled water, 12.5 μl of 2 X Taq Plus Master Mix (Nanjing Vazyme Biotech Co. Ltd, Nanjing, China) and 1 μl of each primer (10 μM). Amplification reactions were run as follows: initial denaturation of 95 °C for 5 min followed by 35 cycles at 95 °C for 30 s, 45 s at 58 °C (annealing temperature) and 1 min at 72 °C with a final extension of 10 min at 72 °C. PCR products were sent to Tianyihuiyuan (Beijing, China) for sequencing with the forward and reverse primers indicated above. AB1 sequence traces were assembled with Sequencher version 5 (Genecodes, Ann Arbor, USA).

### Phylogenetic analyses

The sequences included in this study (Tables 1, 2) were aligned online with MAFFT (https://mafft.cbrc.jp/alignment/server/index.html) using auto strategy, and observed in MEGA 5 (Katoh and Toh 2008). Phylogenetic analyses were based on both maximum likelihood (ML) and Bayesian Inference (BI). RAxML (Stamatakis 2006) and PhyML 3.0 (Guindon et al. 2010) were used for ML analyses. GTRGAMMA was specified as the model of evolution in both programs. The RAxML analyses were run with a rapid Bootstrap analysis (command -f a) using a random starting tree and 1 000 ML bootstrap replicates. The PhyML analyses were implemented using the ATGC bioinformatics platform (available at: http://www.atgcmontpellier.fr/phyml/), with six substitution type and SPR tree improvement, and support obtained from an approximate likelihood ratio test (Anisimova et al. 2011).

For BI, MrBayes was used with a Markov Chain Monte Carlo algorithm incorporating four runs, each consisting of four chains, until the standard deviation of split frequencies was reached. The cold chain was heated at a temperature of 0.25. Substitution model parameters were sampled every 50 generations and trees were saved every 5000 generations. Convergence of the Bayesian analysis was confirmed using AWTY (Nylander et al. 2008) (available at: http://ceb.csit.fsu.edu/awty/). A user-defined tree obtained from the PhyML analyses was used as a starting point for all the Bayesian analyses, which helped to improve convergence of the four runs.

## Results

**Figure 1.**
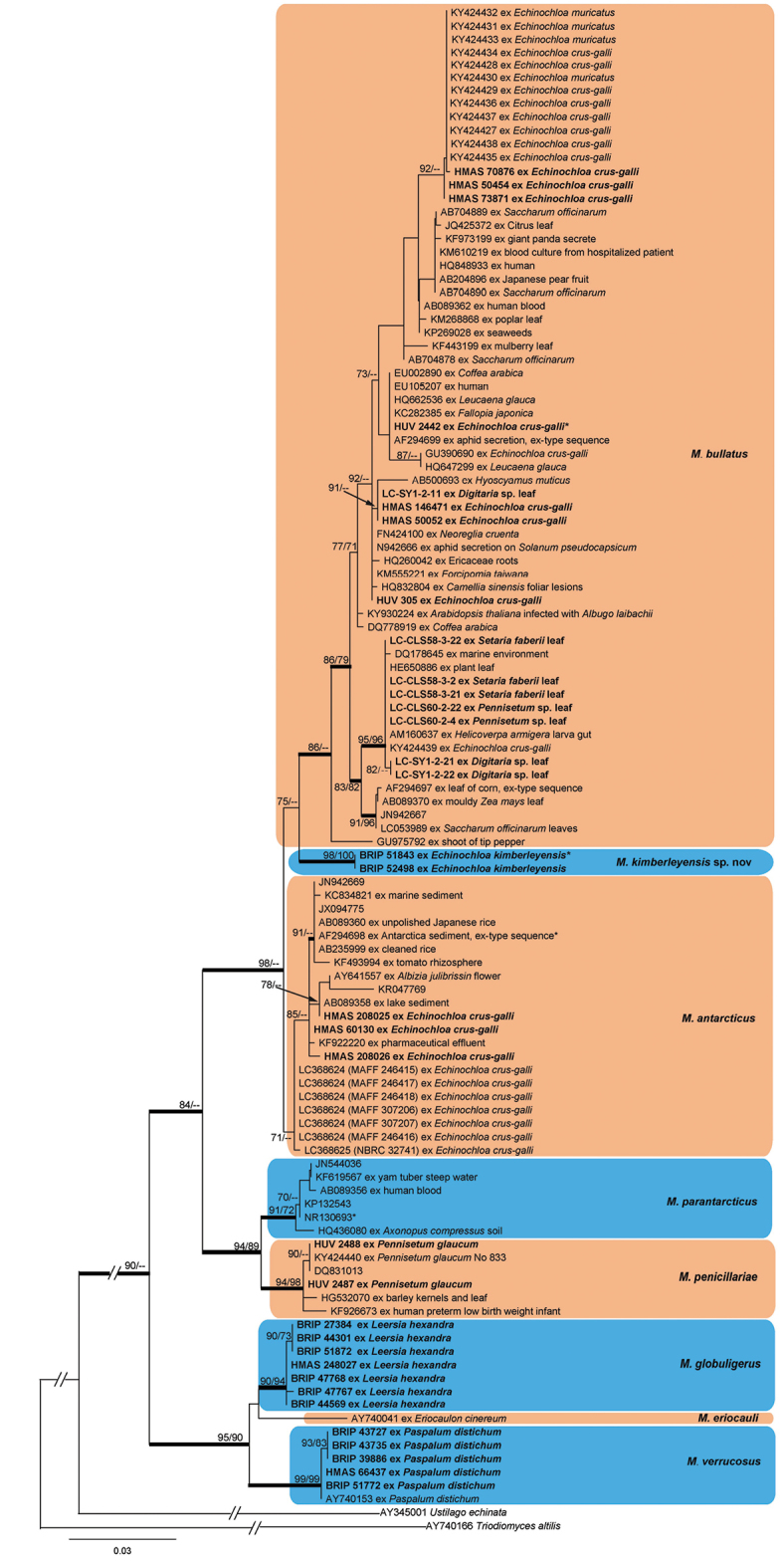
Phylogram obtained from a ML analysis based on the ITS sequence alignment. Values above the branches represent ML bootstrap values (> 70%) from RaxML and PhyML analysis respectively. Thickened branches represent Bayesian posterior probabilities (> 0.95). The scale bar indicates 0.03 expected substitutions per site. * indicates type specimens or type strains.

The ITS dataset comprised the newly sequenced *Moesziomyces* specimens and strains (Table 1) together with the reference sequences of *Moesziomyces* from Kruse et al. (2017) and Tanaka et al. (2019) (Table 2) and *Triodiomycesaltilis* and *Ustilagoechinata* as the outgroup based on the phylogenetic analyses of Wang et al. (2015). The topology of the ML and BI analyses (Fig. 1) were congruent. The phylogenetic analyses revealed eight distinct groups with high support values, including six clades consistent with those recovered by Kruse et al. (2017). The largest clade included specimens of *M.bullatus* on *Echinochloacrus-galli* (the host for the type specimen of *M.bullatus*) and *E.muricata* from Europe, related yeast strains as well as strains formerly assigned to the synonymous species names *Pseudozymaaphidis* and *P.rugulosa* (Kruse et al. 2017). Four well-supported clades comprised teleomorphic specimens on *Echinochloakimberleyensis*, *Leersiahexandra*, *Paspalumdistichum*, and *Pennisetumglaucum* (the latter with related yeast strains). One well-supported clade comprised yeast strains assigned to *M.parantarcticus*. One moderately supported clade comprised teleomorphic specimens on *E.crus-galli* from China and Japan and related yeast strains, assigned to *M.antarcticus*. The remaining single-sequence lineage was formed by *Moesziomyceseriocauli* on *Eriocauloncinereum* (Eriocaulaceae).

**Table 2.** List of *Moesziomyces*, *Triodiomyces*, and *Ustilago* sequences taken from GenBank and used in the phylogenetic analysis.

Species	Source	ITS GenBank accession number	Reference
* Moesziomyces antarcticus *	–	JX094775	Gujjari et al. (unpubl.)
	–	JN942669	An (unpubl.)
	unpolished Japanese rice	AB089360	Sugita et al. 2003
	Antarctica sediment	AF294698	Avis et al. 2001
	*Albiziajulibrissin* flower	AY641557	Wei et al. 2005
	lake sediment	AB089358	Sugita et al. 2003
	tomato rhizosphere	KF493994	Johnston-Monje et al. (unpubl.)
	*Echinochloacrus-galli* sorus	LC368624	Tanaka et al. 2019
	*Echinochloacrus-galli* sorus	LC368624	Tanaka et al. 2019
	*Echinochloacrus-galli* sorus	LC368624	Tanaka et al. 2019
	*Echinochloacrus-galli* sorus	LC368624	Tanaka et al. 2019
	*Echinochloacrus-galli* sorus	LC368624	Tanaka et al. 2019
	*Echinochloacrus-galli* sorus	LC368624	Tanaka et al. 2019
	*Echinochloacrus-galli* sorus	LC368625	Tanaka et al. 2019
* Moesziomyces bullatus *	human preterm low birth weight infant	KF926673	Okolo et al. 2015
	–	DQ831013	Matheny et al. 2006
	Japanese pear fruit	AB204896	Yasuda et al. 2007
	* Saccharum officinarum *	AB704889	Morita et al. 2012
	* Leucaena glauca *	HQ662536	Wei et al. 2011
	human	EU105207	Lin et al. 2008
	human blood	AB089362	Sugita et al. 2003
	human	HQ848933	Xie et al. unpubl.
	* Fallopia japonica *	KC282385	Wang & Liu (unpubl.)
	human blood	KM610219	Bosco-Borgeat & Taverna (unpubl.)
	* Leucaena glauca *	HQ647299	Wei et al. 2011
	* Saccharum officinarum *	AB704890	Morita et al. 2012
	poplar leaf	KM268868	Sun & Yan (unpubl.)
	* Forcipomyia taiwana *	KM555221	Chen (unpubl.)
	seaweed	KP269028	Wang et al. (unpubl.)
	aphid secretion	AF294699	Avis et al. 2001
	* Neoreglia cruenta *	FN424100	Garcia et al. (unpubl.)
	* Saccharum officinarum *	AB704878	Morita et al. 2012
	giant panda secretion	KF973199	Li et al. (unpubl.)
	*Camelliasinensis* leaf lesions	HQ832804	Li et al. (unpubl.)
	* Echinochloa crus-galli *	GU390690	Hamayun & Ahmad (unpubl.)
	aphid secretion on *Solanumpseudocapsicum*	JN942666	An (unpubl.)
	*Citrus* leaf	JQ425372	Soliman (unpubl.)
	–	JN942667	An (unpubl.)
	mouldy *Zeamays* leaf	AB089370	Sugita et al. 2003
	plant leaf	HE650886	Han et al. 2012
	ex-leaf of corn	AF294697	Avis et al. 2001
	* Hyoscyamus muticus *	AB500693	Abdel-Motaal & Itu (unpubl.)
	* Coffea arabica *	EU002890	Vega et al. (unpubl.)
	* Coffea arabica *	DQ778919	Vega et al. 2008
	*Saccharumofficinarum* leaf	LC053989	Surussawadee & Limtong (unpubl.)
	marine environment	DQ178645	Chang et al. 2008
	*Helicoverpaarmigera* larva gut	AM160637	Molnar & Prillinger (unpubl.)
* Moesziomyces bullatus *	marine sediment	KC834821	Qu et al. (unpubl.)
	–	KR047769	Wang et al. (unpubl.)
	pharmaceutical effluent	KF922220	Selvi & Das (unpubl.)
	barley kernels and leaf	HG532070	Korhola et al. 2014
	Ericaceae roots	HQ260042	Walker et al. 2011
	cleaned rice	AB235999	Ikeda et al. 2007
	*Arabidopsisthaliana* infected with *Albugolaibachii*	KY930224	Kruse et al. 2017
	* Echinochloa crus-galli *	KY424439	Kruse et al. 2017
	* Echinochloa crus-galli *	KY424428	Kruse et al. 2017
	* Echinochloa crus-galli *	KY424429	Kruse et al. 2017
	* Echinochloa muricata *	KY424430	Kruse et al. 2017
	* Echinochloa muricata *	KY424431	Kruse et al. 2017
	* Echinochloa muricata *	KY424432	Kruse et al. 2017
	* Echinochloa muricata *	KY424433	Kruse et al. 2017
	* Echinochloa crus-galli *	KY424434	Kruse et al. 2017
	* Echinochloa crus-galli *	KY424435	Kruse et al. 2017
	* Echinochloa crus-galli *	KY424436	Kruse et al. 2017
	* Echinochloa crus-galli *	KY424437	Kruse et al. 2017
	* Echinochloa crus-galli *	KY424427	Kruse et al. 2017
	* Echinochloa crus-galli *	KY424438	Kruse et al. 2017
	shoot of tip pepper	GU975792	Sim et al. (unpubl.)
* Moesziomyces eriocauli *	* Eriocaulon cinereum *	AY740041	Stoll et al. 2005
* Moesziomyces parantarcticus *	–	KP132543	Irinyi et al. 2015
	human blood	AB089356	Sugita et al. 2003
	–	NR130693	An (unpubl.)
	–	JN544036	Chen (unpubl.)
	yam tuber steep water	KF619567	Babajide et al. 2015
	*Axonopuscompressus* soil	HQ436080	Kee & Chia (unpubl.)
* Moesziomyces penicillariae *	* Pennisetum glaucum *	KY424440	Kruse et al. 2017
* Moesziomyces verrucosus *	* Paspalum distichum *	AY740153	Stoll et al. 2005
* Triodiomyces altilis *	* Triodia pungens *	AY740166	Stoll et al. 2005
* Ustilago echinata *	* Phalaris arundinacea *	AY345001	Stoll et al. 2003

## Taxonomy

Based on the phylogenetic analysis and the hosts of the teleomorphs, a new species of *Moesziomyces* is described and another species resurrected. Additionally, the teleomorph of *M.antarcticus* is reported for the first time from China.

### Moesziomyces antarcticus

Taxon classificationFungiUstilaginalesUstilaginaceae

(Goto, Sugiyama & Iizuka) Q.M. Wang, Begerow, F.Y. Bai & Boekhout, Stud. Mycol. 81: 81 (2015)

Figure 2h–k

Sporobolomyces antarcticus Goto, Sugiyama & Iizuka, Mycologia 61: 759 (1969). [Basionym]
Candida antarctica (Goto, Sugiyama & Iizuka) Kurtzman et al. Yeasts: 86 (1983).
Vanrija antarctica (Goto, Sugiyama & Iizuka) R.T. Moore, Bibltheca Mycol. 108: 167 (1987).
Pseudozyma antarctica (Goto, Sugiyama & Iizuka) Boekhout, J. Gen. Appl. Microbiol. 41: 364 (1995).
Trichosporon oryzae H. Ito, Iizuka & T. Sato, Agric. Biol. Chem. 38: 1599 (1974). (synonymy by Q.M. Wang, Begerow, F.Y. Bai and Boekhout).

#### Description.

Sori in scattered ovaries, sometimes deciduous, globose to ovoid, 2–3 mm in length, covered by a smooth green membrane of host tissue origin that becomes brown and ruptures irregularly to expose a granular, black to dark brown mass of spore balls; columella absent. Spore balls variable in shape and size, globose, subglobose, ovoid, elongate to irregular, 130–200 μm in diameter, dark brown, composed of up to several hundred spores, united firmly by fungal sterile cells and spore meshes and wings. Spore globose, ovoid to irregular, slightly polyhedral, (8–) 8.5–9.5 (–10) × (6–) 7–9 (–10) μm, usually with well-developed meshes and wings, subhyaline to pale yellowish-brown; wall 0.5 μm thick, smooth. Some of the sterile cells empty at maturity, thin-walled, with irregular meshes or wings on the spore surface when the spores separates; other sterile cells, globose, ovoid to irregular, slightly polyhedral, (8–) 8.5–9.5 (–10) × (6–) 7–9 (–10) μm, subhyaline to pale yellowish brown; wall 1–1.5 μm thick, smooth.

#### Specimens examined.

CHINA, Sichuan, Chengdu, on *Echinochloacrus-galli*, 15 Sept. 1989, L. Guo leg., HMAS 60130; Guangxi, on *E.crus-galli*, Oct. 2017, R.G. Shivas, M.D.E. Shivas & Y.-M. Li leg., HMAS 208025; Guangxi, on *E.crus-galli*, Oct. 2017, R.G. Shivas, M.D.E. Shivas & Y.-M. Li leg., HMAS 208026.

#### Notes.

The teleomorph of *Moesziomycesantarcticus* was previously reported from Japan, on *Echinochloacrus-galli* (Tanaka et al. 2019). The current report from China, also on *E.crus-galli*, suggests that this smut fungus may be common in the teleomorphic stage, at least in East Asia.

### Moesziomyces globuligerus

Taxon classificationFungiUstilaginalesUstilaginaceae

(Berk. & Broome) Vánky, Bot. Not. 130: 135 (1977)

Figure 2e–g

Thecaphora globuligera Berk. & Broome, Trans. Linn. Soc. London, Bot., Ser. 2, 1: 407 (1880). — Type: AUSTRALIA, Queensland, Brisbane, on Leersiahexandra, F.M. Bailey, No. 86 (K(M) 252436, **lectotype designated here**, MBT 385180, not seen; K(M) 252437, syntype). [Basionym]
Tolyposporium globuligerum (Berk. & Broome) Ricker, J. Mycol. 11:112 (1905).
Testicularia leersiae Cornu, Ann. Sci. Nat. Bot., Sér. 6, 15: 275 (1883).

#### Description.

Sori in some of the ovaries, often deciduous, ellipsoidal to oval, 2.5–4 × 1.5–3 mm, green at first, later brown, smooth, ruptures irregularly to reveal a granular, dark brown mass of spore balls; columella absent. Spore balls subglobose, ellipsoidal or irregular, 75–150 µm in diameter, yellowish brown, composed of up to several hundred spores that separate by moderate pressure. Spores subglobose, ovoid to irregularly polyhedral, (8–) 8.5–11 (–13) × (6–) 7–9 (–10) μm (*xˉ* = 9.6 ± 1.2 × 7.9 ± 0.9 μm, *n* = 50), subhyaline to pale yellowish brown, attached together by multiple narrow cylindrical protuberances about 2 μm wide and 1–2 μm long; wall with irregular meshes and wings, less than 0.5 μm thick, smooth. (Based on specimen BRIP 27384).

#### Specimens examined.

AUSTRALIA, Queensland, Willowbank, on *Leersiahexandra*, 9 Mar. 1998, C. Vánky & K. Vánky leg., BRIP 27384; Queensland, Mareeba, on *L.hexandra*, 1 May 2004, M.D.E. Shivas & R.G. Shivas leg., BRIP 44301; Queensland, Mt Garnet, on *L.hexandra*, 5 May 2005, T.S. Marney & R.G. Shivas leg., BRIP 44569; Northern Territory, Darwin, on *L.hexandra*, 15 Apr. 2008, J. Ray, A.A. Mitchell, A.R. McTaggart & R.G. Shivas leg., BRIP 51872. CHINA, Guangxi province, on *L.hexandra*, Oct. 2017, R.G. Shivas, M.D.E. Shivas, Y.-M. Li, P. Zhao & X.-H. Qi leg., HMAS 248027. THAILAND, Kanchanaburi, on *L.hexandra*, 16 Dec. 2005, R.G. Shivas & M.D.E. Shivas leg., BRIP 47767; Chiang Mai, on *L.hexandra*, 26 Dec. 2005, R.G. Shivas & M.D.E. Shivas leg., BRIP 47768.

#### Notes.

Vánky (1986) considered that *M.globuligerus* was a synonym of *M.bullatus* based on their similar morphologies. Phylogenetic analyses support *M.globuligerus* as a distinct species (Fig. 1), with a teleomorph specific to the pantropical grass *Leersiahexandra* (Berkeley and Broome 1880). The name *Testicularialeersiae* (Cornu 1883), described from infected *Leersiahexandra* in Algeria, is likely a heterotypic synonym of *M.globuligerus*, but this has not been checked by molecular phylogenetic analysis. The type material of *Thecaphoraglobuligera* was collected circa 1878 from near the Brisbane River, Queensland, Australia by the botanist F. M. Bailey (Berkeley and Broome 1880). Original material of this specimen (F.M. Bailey, No. 86) could not be found in the Australian herbaria BRI and BRIP, where most of F.M. Bailey’s specimens are held. Two syntypes were located in K(M), of which K(M) 252436 ex C.E. Broome herbarium (BM) was selected as lectotype of *T.globuligera* (now *M.globuligerus*). The material in the second specimen, K(M) 252437 from the Berkeley herbarium, was scant (Dr Begoña Aguirre-Hudson pers. comm).

**Figure 2.**
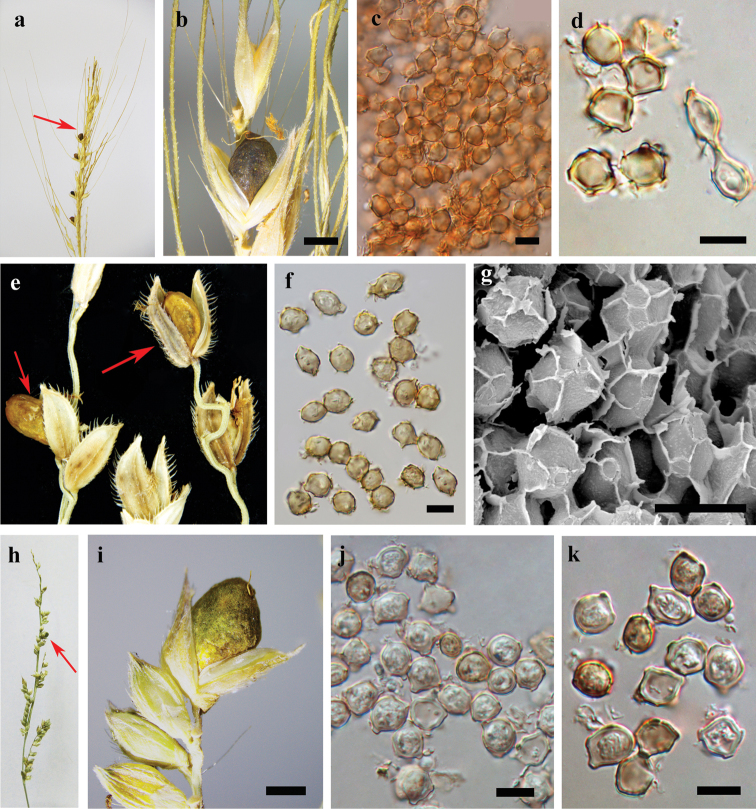
**a–d***Moesziomyceskimberleyensis* (holotype BRIP 51843) **e–g***Moesziomycesglobuligerus* (BRIP 27384) **h–k***Moesziomycesantarcticus* (HMAS 60130). a, b: sori. c, d, f, j, k: spores under LM. g: spores under SEM. Scale bars: 1 mm (**b, i**); 10 µm (**c, d, f, g, j, k**).

### Moesziomyces kimberleyensis

Taxon classificationFungiUstilaginalesUstilaginaceae

Y.M. Li, L. Cai & R.G. Shivas sp. nov.

827986

Figure 2a–d

#### Type.

AUSTRALIA, Western Australia, Kununurra, Mulligan’s Lagoon Road, on *Echinochloakimberleyensis*, 9 Apr. 2008, A.R. McTaggart, V.L. Challinor, A.D.W. Geering, M.D.E. Shivas & R.G. Shivas leg. (holotype: BRIP 51843).

#### Etymology.

Named after the Kimberley region of northern Western Australia from where it was collected.

#### Description.

Sori in some of the ovaries, often deciduous, globose to ovoid, 3–6 × 2–4 mm, green at first, later brown, smooth, ruptures irregularly to reveal a granular, dark brown mass of spore balls; columella absent. Spore balls subglobose, ovoid, elongate or irregular, 275–100 µm diam, dark brown, composed of up to several hundred spores, separated by moderate pressure. Spore globose, ovoid to irregular, slightly polyhedral, (9–) 9.5–12 (–14.5) × (8–) 8.5–9.5 (–10) μm (*xˉ* = 10.5 ± 1.2 × 8.9 ± 0.7 μm, *n* = 50), subhyaline to yellowish brown, attached together by multiple narrow cylindrical protuberances about 2 μm wide and 1–2 μm long; wall with irregular meshes and wings, 0.5 μm thick, smooth.

#### Additional specimen examined.

AUSTRALIA, Western Australia, Kununurra, Mulligan’s Lagoon Road, on *E.kimberleyensis*, 7 May 2009, A.R. McTaggart, M.J. Ryley, M.D.E. Shivas & R.G. Shivas leg. (BRIP 52498).

#### Notes.

*Moesziomyceskimberleyensis* was shown in the phylogenetic analysis to reside in a well-supported clade sister to *M.bullatus*. *Moesziomyceskimberleyensis* is only known from the teleomorph, which forms sori in flowers of *E.kimberleyensis*, and thereby differs from *M.bullatus* by host association. *Moesziomyceskimberleyensis* is only known from one location in Western Australia on *E.kimberleyensis*, which is an endemic grass in the tropical and subtropical woodlands of northern Australia.

## Discussion

The phylogenetic analyses in this study supported the host specificity of the teleomorphic stage of six species of *Moesziomyces*, specifically, *M.antarcticus* on *Echinochloacrus-galli*, *M.bullatus* on *E.crus-galli* and *E.muricata*, *M.globuligerus* on *Leersiahexandra*, *M.kimberleyensis* on *E.kimberleyensis*, *M.penicillariae* on *Pennisetumglaucum*, and *M.verrucosus* on *Paspalumdistichum*. The teleomorph of *M.eriocauli* may be specific to *Eriocaulon* spp., although this cannot be ascertained from the sequence data of one specimen. Specimens that have been assigned to *M.bullatus* were not well resolved and formed a number of smaller clades with varying degrees of support (Fig. 1). The *M.bullatus* clade contained several anamorphic yeasts isolated from diverse habitats (Wang et al. 2015; Kruse et al. 2017), including leaves of *Digitaria* sp., *Pennisetum* sp., and *Setariafaberii*. This shows that the anamorphs of *Moesziomyces* are widespread in the environment as saprobes.

The anamorphs of *Moesziomyces*, together with most members of the Ustilaginales, have a dimorphic lifecycle comprised of a parasitic dikaryotic phase characterized by teliospores, together with a saprobic yeast-like haploid phase (Brefeld 1883; de Bary 1884; Sampson 1939; Begerow et al. 2014). The teliospores are generally thick-walled and darkened, which protects against desiccation and UV radiation, thereby facilitating survival and long-distance dispersal (Piepenbring et al. 1998). The basidiospores are usually thin-walled, hyaline, and survive as free-living saprobic yeasts that may occur on a vast diversity of substrates (Wang et al. 2015; Kruse et al. 2017; Tanaka et al. 2019). There is genomic evidence that some saprobic ustilaginalean yeasts, e.g. *M.antarcticus*, *Kalmanozymabrasiliensis* (= *P.brasiliensis*), *Pseudozymahubeiensis*, and the yeast stage of *M.bullatus* (= *P.aphidis*), have retained the capacity to produce effector proteins, which hints at the possibility that undiscovered plant pathogenic stages may exist for these fungi (Sharma et al. 2018). Indeed, a teleomorph for *M.antarcticus* (=*P.antarctica*) was recently reported for the first time on *Echinochloacrus-galli* (Tanaka et al. 2019). Further collections are needed to resolve the ecological relationships and elucidate the life cycles of the ustilaginalean fungi and their hosts.

## Supplementary Material

XML Treatment for Moesziomyces antarcticus

XML Treatment for Moesziomyces globuligerus

XML Treatment for Moesziomyces kimberleyensis
